# Reactivation of variably sealed joints and permeability enhancement in geothermal reservoir rocks

**DOI:** 10.1186/s40517-023-00271-5

**Published:** 2023-11-13

**Authors:** Alexandra R. L. Kushnir, Michael J. Heap, Patrick Baud, Thierry Reuschlé, Jean Schmittbuhl

**Affiliations:** 1 Present Address: Rock Physics and Geofluids Laboratory (RPGL), IIC, ENAC, École polytechnique fédérale de Lausanne, Station 18, CH-1015 Lausanne, Switzerland; 2 Insitut Terre et Environnement de Strasbourg, UMR 7063, Université de Strasbourg, CNRS, 5 Rue René Descartes, 67084 Strasbourg, France; 3 Institut Universitaire de France (IUF), Paris, France

**Keywords:** Buntsandstein, Upper Rhine Graben, Variably filled joints, Rock physical properties, Permeability, Rock strength

## Abstract

Hydraulic stimulation of enhanced deep geothermal reservoirs commonly targets pre-existing joint networks with the goal of increasing reservoir permeability. Here, we study the permeability and strength of joint-free and jointed Buntsandstein sandstones from the EPS-1 exploratory borehole at the Soultz-sous-Forêts geothermal site (France). The studied jointed samples contain naturally formed fractures that are variably filled with secondary mineralisation. We find that the permeability of these rocks is more sensitive to the presence and orientation of bedding than to the presence of joints at the scale of the samples: permeability is lowest in samples where bedding is oriented perpendicular to the direction of fluid flow. While well-sealed joints can act as barriers to fluid flow, partially filled joints neither inhibit nor promote fluid flow with respect to their joint-free counterparts. These samples were then deformed under triaxial conditions to assess (1) whether deformation reactivates pre-existing joints, and (2) how permeability changes as a result of deformation. We find that the mechanical response of the rocks depends on the extent to which joints are sealed. Well-sealed joints locally increase rock strength and experimentally induced fractures do not exploit pre-existing joint surfaces; partially sealed joints, by contrast, act as planes of weakness that localise strain. Although the permeability of all samples increased during deformation, permeability increase was largest in samples with poorly filled joints. We conclude that hydraulic stimulation operations must carefully consider the extent to which targeted joint networks are filled. Partially sealed joints are ideal targets for stimulation: these features act as planes of weakness within the rock mass and their reactivation can result in significant increases in permeability. By contrast, well-sealed joints may increase rock strength locally and may never reactivate during stimulation, making them poor targets for permeability enhancement.

**Supplementary Information:**

The online version contains supplementary material available at 10.1186/s40517-023-00271-5.

## Introduction

Geothermal systems offer an attractive renewable energy resource that is available year-round and can be developed and managed locally, thereby contributing to regional energy independence. While high enthalpy, natural steam geothermal systems have an established foothold in several countries (Barbier 2002), these resources are geographically restricted to tectonically active regions. The successful development of a number of deep geothermal resources has demonstrated the viability of exploiting deep (in excess of 1 km) crustal reservoirs for power generation (Lu 2018) and direct-use (Lund and Toth 2021), expanding the typical reach of this energy resource to intra-continental regions. While deep geothermal energy has the potential to provide significant base load energy (e.g., Tester et al. 2007), technical challenges to reservoir development remain a key stumbling block to widespread adoption (McClure and Horne 2014; Clauser and Ewert 2018).

The efficient operation of geothermal systems depends on the continuous movement of hot hydrothermal fluids within the reservoir, which is often facilitated by large joint networks (Moeck 2014). Enhanced Geothermal Systems (EGS) artificially increase the permeability, and thus the productivity, of existing geothermal resources using reservoir stimulation techniques (Clauser 2006; Huenges and Ledru 2011). These methods target existing—often mineral-sealed—joint systems through chemical, hydraulic, and thermal stimulation and the success of these methods depends on the efficacy with which they are able to increase reservoir permeability. Each stimulation technique targets a different property of the joint network: chemical stimulation aims to remove mineral scaling from joint spaces (Portier et al. 2009; Na et al. 2016; Nami et al. 2008); thermal stimulation relies on the contrast in thermal expansion between rock components to generate stresses that create or reactivate joints (Kumari et al. 2018; Sutra et al. 2017); and hydraulic stimulation uses moderate increases in injection pressure to mechanically reactivate existing joints and, under certain circumstances, create new fractures (Xie and Min 2016; Gischig et al. 2020; Li et al. 2022).

Shear stimulation (or hydroshearing) can induce shear displacement on existing joint surfaces—propping joints open on pre-existing asperities and at relatively low injection pressures (Evans et al. 2005; Willis-Richards et al. 1996)—while also propagating new fractures within the system (Moeck et al. 2009; McClure and Horne 2014). However, fluid injection pressures need to be carefully managed during hydraulic stimulation to account for in situ stresses and reduce the potential for induced seismicity (Deichmann and Giardini 2009). Shear stimulation modelling can be used to predict the conditions under which joints can reactivate but these approaches often assume that joints act as inherent planes of weakness within a rock mass and often do not account for the role of joint-filling mineralisation (Xie and Min 2016; Fu et al. 2013; Settgast et al. 2017). Indeed, while the reactivation of open fractures has been well studied (e.g., Esaki et al. 1999; Samuelson et al. 2009; Yildirim et al. 2020; Hutka et al. 2023), the response of sealed fractures to reactivation has received relatively little attention (e.g., Ye et al. 2020).

In this study, we experimentally assess the role of variably mineral-sealed joints on the permeability and strength of Buntsandstein sandstone from the EPS-1 exploratory borehole at the Soultz-sous-Forêts geothermal site (France). We deform joint-free and jointed sandstones in triaxial compression to better understand the ease with which sealed joints can be reactivated and to what extent joint reactivation can increase rock permeability, with implications for stimulation strategies at EGS reservoirs.

## Experimental materials

### The Upper Rhine Graben and the Soultz-sous-Forêts geothermal site (France)

Geothermal exploitation in the Upper Rhine Graben targets thermal anomalies created by kilometre-scale hydrothermal convection cells located within the region’s fractured granite basement and overlying sedimentary sequences (Pribnow and Schellschmidt 2000; Baillieux et al. 2013). The Paleozoic granite is often the target of geothermal exploitation (e.g., at Soultz-sous-Forêts and Rittershoffen in France; Gérard et al. 2006; Baujard et al. 2017) and these rocks are well characterised (e.g., Dezayes et al. 2010; Ledesert et al. 2010; Surma and Géraud 2003). While the geothermal reservoirs developed at Soultz-sous-Forêts and Rittershoffen are below 3 km depth (Gérard et al. 2006; Baujard et al. 2017), temperatures in excess of 100 °C are observed at 1 km depth in the Permo-Triassic sediments, including the Buntsandstein (Cuenot et al. 2008). These shallower depths may offer an economically attractive alternative to the deep Paleozoic granite. Indeed, the geothermal potential of the Permo-Triassic sediments has been demonstrated at Bruchsal (Herzberger et al. 2010) in Germany and Cronenbourg (Housse 1984) in France, while a multi-horizon concept exploiting the transition from the Permo-Triassic sandstone to Paleozoic granite has been explored at Landau (Hettkamp et al. 2007) and Insheim (Baumgärtner et al. 2013) in Germany and Rittershoffen, France (Vidal et al. 2017).

Numerical modelling has shown that to maintain regional convection of hot fluids in the Upper Rhine Graben, the equivalent permeability of the Permo-Triassic sedimentary sequences must be maintained above a threshold permeability of at least 10^–15^ m^2^ (Guillou-Frottier et al. 2013; Magnenet et al. 2014). Critically, laboratory measurements of the matrix permeability of the majority of the Buntsandstein (10^−19^ to 10^−13^ m^2^; Heap et al. 2017) is well below this threshold and geothermal circulation in the region is reliant on the existence of extensive networks of open, pervasively connected joints (Genter et al. 1997; Haffen et al. 2013; Vidal and Genter 2018) that increase the equivalent permeability of the Buntsandstein by several orders of magnitude. While joint networks are observed in the Buntsandstein, secondary mineral precipitation is widespread (Vidal and Genter 2018) necessitating anthropogenic stimulation to maintain reservoir productivity.

### Rock description and microstructure

We have selected three Buntsandstein sandstones from different depths of the EPS-1 exploratory borehole located near Soultz-sous-Forêts, France (Genter and Traineau 1996). These rocks come from the *Couches Intermédiaires* (Upper Buntsandstein, 1021 to 1048 m b.s.l.), the *Couches de Karlstal* (Middle Buntsandstein, 1048 to 1160 m b.s.l.), and the *Couches de Trifels* (Middle Buntsandstein, 1256 to 1349 m b.s.l.). At each of these three sampling depths, borehole cores (78 mm in diameter) of joint-free (i.e., free of naturally formed fractures on the borehole scale) and jointed (i.e., containing naturally formed fractures) sandstones were selected.

**Fig. 1:**
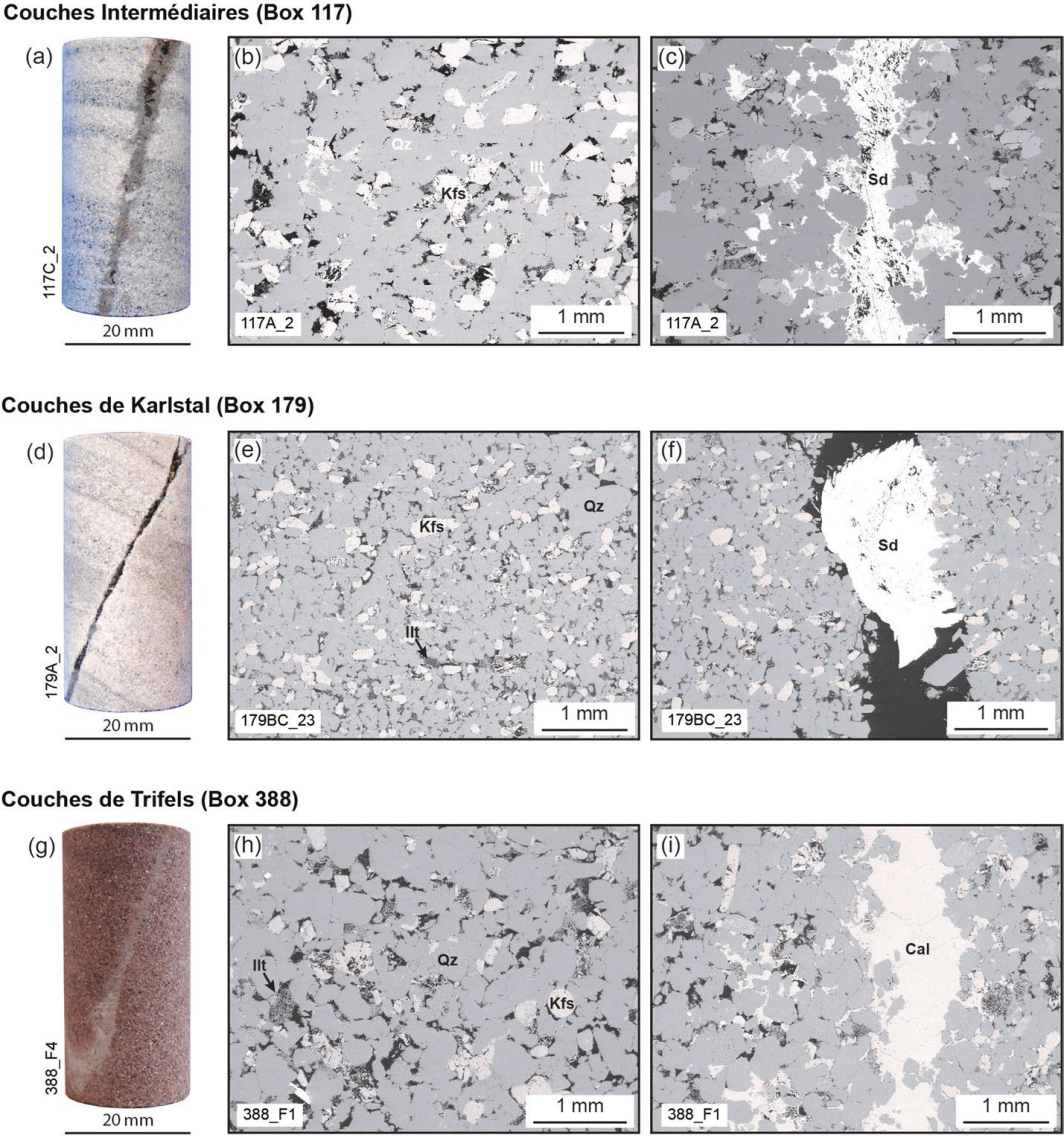
Pre-deformation microstructure. Photographs of samples prior to deformation (**a**, **d**, **g**), showing the extent of secondary mineral precipitation in naturally formed joints. Scanning electron microscope (SEM) images of the matrix (**b**, **e**, **h**) and joints (**c**, **f**, **i**) of the studied sandstones. **a**, **b**, **c** *Couches Intermédiaires* (Box 117); **d**, **e**, **f** *Couches de Karlstal* (Box 179); **g**, **h**, **i** *Couches de Trifels* (Box 388). *Qz*: quartz, *Kfs*: K-feldspar, *Cal*: calcite, *Sd*: siderite, *Ilt*: illite

**Fig. 2:**
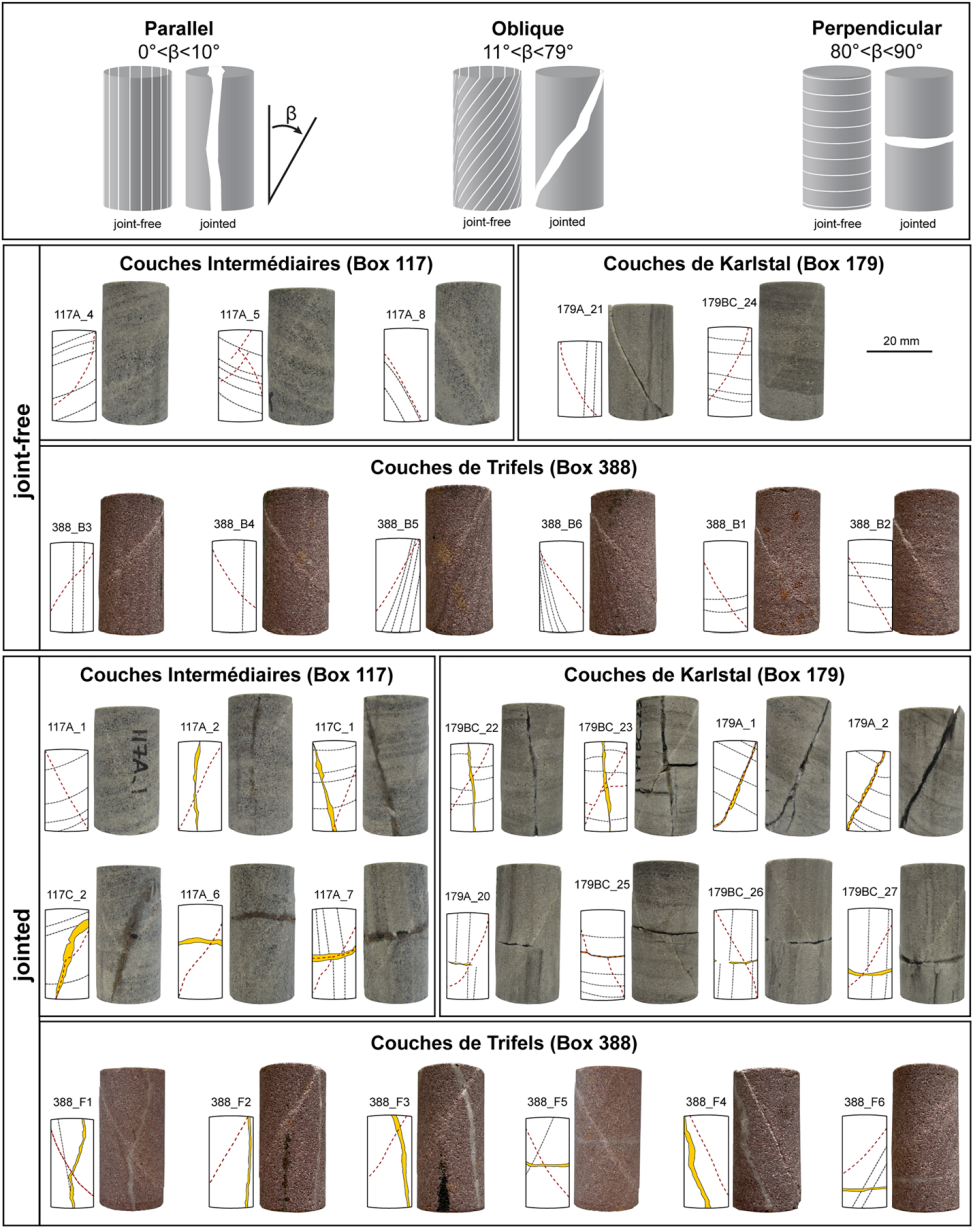
Photographs of all samples after deformation. Top panel provides schematics of idealised samples containing bedding or joints oriented parallel, oblique, and perpendicular to the longitudinal axis of the cores. The bottom two panels contain photographs and sketches of joint-free and jointed samples, deformed to failure in triaxial compression. Dotted lines denote bedding; yellow bands denote joints; and dashed red lines denote experimentally induced failure planes

The *Couches Intermédiaires* are braided fluvial sandstones (Vernoux et al. 1995) and the samples used here (depth of 1038 m; box number 117) are light to dark grey (Fig. 1a). The bedded rock matrix contains quartz and K-feldspar grains up to 0.5 mm in diameter, with some K-feldspar grains having experienced alteration to illite (Fig. 1b). The matrix does not contain microfractures and bedding is defined by changes in matrix porosity. Where present, joints are up to 5 mm wide (average width: 1.7 mm) and moderately filled by siderite: porosity within the joint space presents as angular voids and joint-filling material is observed in the pore space of the matrix adjacent to the joints (Fig. 1c). Beds are displaced by 5 to 10 mm along the joint surfaces (Figs. 1a, 2) and, in some places, joints pinch out on the sample scale (Fig. 2).

The *Couches de Karlstal* are light brown to grey bedded aeolian sandstones (Duringer et al. 2019) and the samples used here (depth of 1089 m; box number 179) are finely bedded (Fig. 1d). The sandstone matrix is free of microfractures, and beds are defined by alternating 1 to 2 mm-thick layers of low- and high-porosity bands. The matrix is composed of quartz and K-feldspar grains ranging from 0.1 to 0.5 mm in diameter, with some K-feldspar grains having been altered to illite (Fig. 1e). Where present, joints are up to 6.5 mm wide (average width: 0.6 mm) and poorly sealed (i.e., there is significant porosity within the joint volume, Fig. 1f) by siderite. As in the *Couches Intermédiaires* samples, beds are displaced by 5 to 10 mm along the joint surfaces and some joints pinch out on the sample scale (Fig. 2).

The *Couches de Trifels* are dark pink to red fluvial sandstones (Duringer et al. 2019; Fig. 1g). At 1277 m depth (box number 388), these rocks are weakly bedded and contain quartz and K-feldspar grains that are 0.5 mm in diameter, with some K-feldspar grains having experienced alteration to illite (Fig. 1h). Rare beds occur as 1 to 3 mm-thick layers in an otherwise homogeneous and unfractured groundmass and are defined by changes in porosity. Naturally occurring joints are 0.3 to 3 mm wide (average width: 1.1 mm) and generally well sealed with calcite: porosity within the joint space is limited to microcracks and joint-filling material is observed in the pore space of the matrix adjacent to the joints (Fig. 1i).

### Sample preparation

To constrain the role of the dominant structural feature—bedding or joint—orientation, $\beta$, 31 cylindrical samples were cored such that the dominant feature was inclined approximately parallel ($0^\circ <\beta <10^\circ$), perpendicular ($80^\circ <\beta <90^\circ$), or oblique ($11^\circ <\beta <79^\circ$) to the longitudinal axis of the sample (Fig. 2, top panel; Table 1). Due to the limited quantity of borehole material, we were only able to produce 9 samples of *Couches Intermédiaires* (3 bedded, 6 jointed), 10 samples of *Couches de Karlstal* (2 bedded, 8 jointed), and 12 samples of *Couches de Trifels* (6 bedded, 6 jointed). Where possible, two samples were prepared for each bedding and joint orientation (e.g., 2 oblique, 2 parallel, and 2 perpendicular; Table 1, Fig. 2). All samples were cored to a diameter of 20 mm and precision-ground to a nominal length of 40 mm, such that the length to diameter ratio of each sample was two. Samples were then washed with deionised water and dried under vacuum at 40 °C for at least 48 h. A selection of the *Couches Intermédiaires* and *Couches de Karlstal* samples used in this study have been previously described and measured by Griffiths et al. (2016) (see Table 1). For clarity, we refer to samples without joints as “joint-free” and samples containing variably filled joints as “jointed”.

**Table 1:** Summary of the physical properties of the joint-free and jointed Buntsandstein sandstones measured in this study

Sample	Length (mm)	Diameter (mm)	Bedding dip (°)	Joint dip (°)	Average joint thickness (mm)	Range of joint thickness (mm)	Dry bulk density (g/cm^3^)	Initial connected porosity	k_i_ (m^2^)	k_f_ (m^2^)	Peak differential stress (MPa)	Young’s modulus (GPa)	Fracture dip (°)
Couches Intermédiaires (Box 117)													
Joint-free samples													
117A_4*	40.00	20.12	75	–	–	–	2.37	0.096	5.28 × 10^–17^	2.45 × 10^–15^	144.0	23	31
117A_5*	38.63	19.87	67	–	–	–	2.42	0.082	1.07 × 10^–16^	8.77 × 10^–15^	145.0	28	–
117A_8*	40.09	20.12	29	–	–	–	2.34	0.109	9.43 × 10^–17^	2.20 × 10^–16^	144.6	26	30
Jointed samples													
117A_1*	36.37	20.14	68	7	2.5	0–4.6	2.43	0.093	5.83 × 10^–17^	2.78 × 10^–15^	134.6	17	26
117A_2*	40.33	20.15	–	2	0.7	0–1.2	2.44	0.098	8.54 × 10^–17^	9.10 × 10^–16^	158.2	29	23
117A_6*	40.43	20.15	–	89	0.7	0.8–2.0	2.41	0.099	6.47 × 10^–17^	6.94 × 10^–16^	165.3	27	35
117A_7*	40.67	20.15	4	86	1.8	0.8–3.4	2.43	0.092	5.81 × 10^–17^	5.74 × 10^–16^	153.2	21	31
117C_1	40.10	19.93	78	14	2.3	0.8–4.2	2.49	0.094	2.02 × 10^–17^	1.06 × 10^–15^	147.4	19	21
117C_2	40.05	19.93	78	16	2.4	0.3–5.1	2.45	0.096	2.25 × 10^–17^	7.55 × 10^–16^	156.7	25	16
Couches de Karlstal (Box 179)													
Joint-free samples													
179A_21*	36.02	20.16	3	–	–	–	2.38	0.100	1.77 × 10^–16^	3.28 × 10^–14^	136.3	19	29
179BC_24*	40.11	20.14	79	–	–	–	2.37	0.108	2.99 × 10^–17^	6.61 × 10^–16^	130.6	20	28
Jointed samples													
179A_1	39.93	19.92	52	23	0.7	0.2–1.4	2.39	0.102	7.10 × 10^–17^	9.30 × 10^–15^	89.0	24	23
179A_2	40.07	19.93	61	28	0.8	0.1–1.7	2.34	0.122	2.92 × 10^–16^	1.35 × 10^–13^	67.8	23	28
179A_20*	40.45	20.18	5	84	0.4	0–6.2	2.37	0.104	1.89 × 10^–16^	7.52 × 10^–16^	128.5	18	30
179BC_22*	40.05	20.15	90	3	0.6	0–1.0	2.40	0.100	4.58 × 10^–17^	1.52 × 10^–16^	121.0	18	32
179BC_23*	40.93	20.16	90	5	0.9	0–1.7	2.36	0.111	1.36 × 10^–16^	2.00 × 10^–15^	120.6	22	31
179BC_25*	39.94	20.14	90	86	0.3	0.1–0.6	2.37	0.107	2.52 × 10^–17^	3.02 × 10^–17^	130.0	21	22
179BC_26*	40.60	20.16	90	6	0.4	0–1.0	2.33	0.120	8.58 × 10^–17^	3.08 × 10^–15^	120.9	23	29
179BC_27*	40.40	20.16	0	90	0.9	0–1.7	2.36	0.108	1.29 × 10^–16^	2.75 × 10^–16^	104.0	20	27
Couches de Trifels (Box 388)													
Joint-free samples													
388_B1	41.42	20.04	90	–	–	–	2.28	0.142	2.14 × 10^–16^	2.66 × 10^–15^	115.4	22	33
388_B2	41.46	20.06	90	–	–	–	2.28	0.148	1.03 × 10^–16^	4.85 × 10^–16^	115.2	21	28
388_B3	40.93	20.06	5	–	–	–	2.27	0.152	4.68 × 10^–16^	8.75 × 10^–16^	113.5	22	39
388_B4	41.18	20.05	1	–	–	–	2.26	0.155	5.63 × 10^–16^	8.32 × 10^–16^	112.4	22	29
388_B5	41.28	20.05	21	–	–	–	2.29	0.145	2.11 × 10^–16^	5.23 × 10^–16^	111.8	19	30
388_B6	40.77	20.05	15	–	–	–	2.29	0.144	2.08 × 10^–16^	3.62 × 10^–16^	103.2	16	39
Jointed samples													
388_F1	41.49	20.03	16	6	0.9	0.5–1.9	2.32	0.134	3.55 × 10^–16^	6.68 × 10^–16^	117.9	19	35
388_F2	41.44	20.05	–	0	0.9	0.3–1.5	2.30	0.138	5.08 × 10^–16^	1.13 × 10^–15^	120.8	22	34
388_F3	41.10	20.04	14	22	1.4	0.7–2.2	2.31	0.136	6.66 × 10^–16^	1.02 × 10^–15^	129.3	21	31
388_F4	40.96	20.03	–	20	1.8	1.0–2.9	2.30	0.139	7.63 × 10^–16^	2.59 × 10^–15^	129.6	20	26
388_F5	41.21	20.06	26	90	0.8	0.4–1.3	2.27	0.151	1.28 × 10^–16^	1.43 × 10^–15^	116.6	19	34
388_F6	41.15	20.04	30	86	0.8	0.6–1.2	2.27	0.152	2.81 × 10^–16^	4.33 × 10^–16^	119.5	22	33

^*^denotes samples also studied by Griffiths et al. (2016); bedding/joint/fracture orientations are given in degrees and measured from the sample longitudinal axis (see Fig. 2); *k*_*i*_ is pre-deformation permeability; *k*_*f*_ is post-deformation permeability

Joint, bedding, and fracture orientations were measured on sample photographs (Table 1). To do this, samples were photographed such that the strike of the joint, bedding, or experimentally induced failure plane was orientated into the plane of the image and the angle of dip of the feature was measured using the angle measurement feature in Adobe Photoshop. The samples in this study were not oriented with respect to the borehole core and, therefore, were not oriented with respect to the geothermal reservoir. Consequently, feature orientation is given only by the dip of the feature plane. The variability on any given angle measurement is ± 1°. Joint thicknesses were also measured on sample photographs using the ruler tool in Adobe Photoshop (Table 1). The average joint thickness was calculated using ten measurements along the visible joint length; minimum and maximum joint thicknesses were measured at the narrowest and widest widths observed, respectively. We highlight that the joint thicknesses given herein are based on surface observations of the samples and do not account for thickness variability within the samples. The variability on any given thickness measurement is ± 0.1 mm.

## Experimental methods

### Density and porosity

The dry bulk density,$\rho$, of all samples was calculated using the sample mass, *m*, and geometric bulk volume, *V*_*b*_: $\rho =\frac{m}{{V}_{b}}$. The skeletal volume of all dried, cylindrical samples was measured using a helium pycnometer (Micromeritics AccuPyc II 1340). Connected gas porosity,${\phi }_{gas}$, was calculated using the geometric bulk volume of the sample and the volume of the solid rock matrix and any isolated void space, *V*_*s*+*i*_:${\phi }_{gas}=\left(1-\frac{{V}_{s+i}}{{V}_{b}}\right)$.

### Permeability

We measured the permeability of each sample (1) before deformation (hereafter referred to as the pre-deformation permeability, *k*_*i*_) and (2) after deformation (hereafter referred to as the post-deformation permeability, *k*_*f*_). Permeability was measured along the sample length using a benchtop nitrogen gas permeameter (described by Farquharson et al. 2016) under steady-state flow (for *k* > 10^–17^ m^2^) or transient pulse (for *k* < 10^–17^ m^2^) conditions. All measurements were made under a confining pressure of 1 MPa and at ambient laboratory temperature. The data were assessed for fluid flow artifacts due to turbulent flow and gas slippage using the Forchheimer and Klinkenberg corrections, respectively. A full description of the methods and application of these fluid flow corrections can be found in Kushnir et al. (2018).

### Triaxial deformation

Once the connected porosity and permeability of the samples were determined, each sample was wrapped in a single layer of thin (< 1 mm-thick) copper foil and saturated under vacuum with deionised and de-aired water. Water-saturated samples were then loaded into a triaxial rock deformation press at the Strasbourg Institute for Earth and the Environment (ITES), described by Klein and Reuschlé (2003).

The principal vertical stress at Soultz-sous-Forêts can be calculated ${S}_{v}=0.024z$, where *z* is in metres and stress is in MPa (Rummel and Baumgärtner 1992); the pore fluid pressure at depth is ${P}_{p}=0.9+0.0098z$, where *z* is in metres and pore pressure is in MPa (Valley and Evans 2007). Assuming a simple effective stress law, the effective confining pressure (*S*_*v*_*-P*_*p*_) at the depths considered in this study (1038 to 1277 m) is between 13.8 MPa and 17.2 MPa and, for the purposes of this study, we have chosen to run all triaxial experiments at an effective confining pressure, *P*_*eff*_, of 14.5 MPa.

Samples were subjected to a confining pressure of 15 MPa and a pore fluid pressure of 0.5 MPa and the sample microstructure was allowed to equilibrate to these conditions for a minimum of 12 h. All samples were then deformed axially at a constant strain rate of 10^−6^ s^−1^. Confining pressure and pore fluid pressure were held constant during deformation using servo-controlled pumps equipped with encoders that monitored changes in volume. During deformation, axial displacement and axial load were monitored using a linear variable differential transducer (LVDT) and a pressure transducer that monitored the pressure inside the axial pressure circuit, respectively. These values were then converted to axial strain and axial stress. The static Young’s modulus of all samples is taken as the slope of the apparently linear portion of the stress–strain curve, where the samples behave elastically.

After deformation, samples were slowly unloaded, removed from the triaxial press, dried under vacuum at 40 °C for a minimum of 48 h, and their post-deformation permeabilities were measured. The copper foil was subsequently removed, the samples were photographed, and the orientations of the experimentally induced failure planes were measured.

## Results

All data are provided in Table 1 and the Additional file 1. These data are subject to measurement uncertainties inherent to the experimental equipment used. Overall, the uncertainty on all the data measured experimentally is below 1% and the resulting error bars are significantly smaller than the symbol sizes used in all figures in this study.

### Pre-deformation connected porosity and permeability

**Fig. 3:**
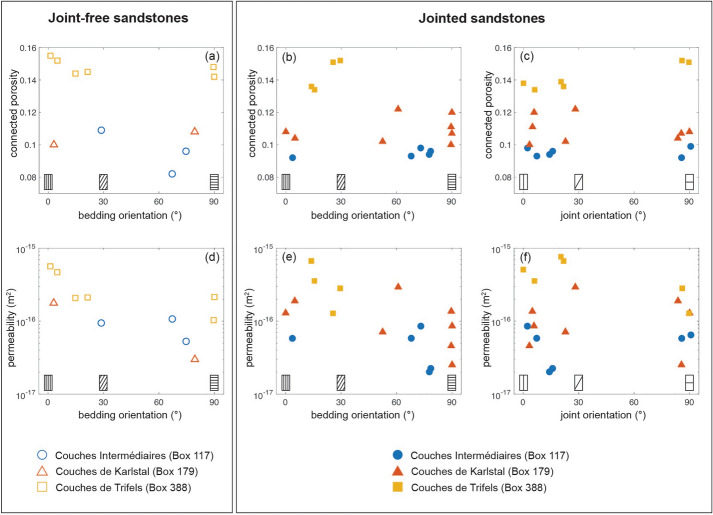
Pre-deformation connected porosity and permeability of the Buntsandstein sandstones as a function of bedding and joint orientation. Joint-free samples are shown in the left panel (unfilled symbols); jointed samples are shown in the right panel (filled symbols). Circles denote *Couches Intermédiaires* (Box 117) samples; triangles denote *Couches de Karlstal* (Box 179) samples; squares denote *Couches de Trifels* (Box 388) samples. **a**, **b**, **c** Connected porosity. **d**, **e**, **f** Permeability. Measurement error is within symbol size

The connected porosity of all samples is between 0.08 and 0.16 and there is no significant difference between the connected porosities of joint-free or jointed samples (Table 1). The average connected porosity of the *Couches Intermédiaires, Couches de Karlstal,* and *Couches de Trifels* sandstones are 0.095, 0.108, and 0.145, respectively. There is no systematic relationship between connected porosity and bedding orientation (Fig. 3a, b), nor between connected porosity and joint orientation (Fig. 3c). We do not observe a systematic change in connected porosity as a function of joint thickness (Additional file 1).

The pre-deformation permeability, *k*_*i*_, of all samples is between 2 × 10^−17^ and 8 × 10^−16^ m^2^ (Table 1). The average pre-deformation permeability of the joint-free *Couches Intermédiaires*, *Couches Karlstal*, and *Couches de Trifels* sandstones are 8.5 × 10^−17^ m^2^, 7.7 × 10^−17^ m^2^, and 3.0 × 10^−16^ m^2^, respectively. Jointed *Couches Intermédiaires, Couches Karlstal,* and *Couches de Trifels* sandstones have an average pre-deformation permeability of 5.2 × 10^−17^ m^2^, 1.4 × 10^−16^ m^2^, and 4.5 × 10^−16^ m^2^, respectively. Overall, permeability increases as a function of increasing connected porosity: *Couches Intermédiaires* sandstones are the least permeable and the *Couches de Trifels* sandstones are the most permeable (Additional file 1).

The pre-deformation permeability of both joint-free and jointed sandstones decreases by approximately an order of magnitude as bedding orientation approaches 90° (i.e., perpendicular to the sample length and thus, fluid flow direction; Fig. 3d, e). While there is no systematic relationship between permeability and joint orientation in *Couches Intermédiaires* and *Couches de Karlstal* samples (Fig. 3f), the *Couches de Trifels* sandstones containing joints oriented perpendicular to fluid flow direction (i.e., $80^\circ <\beta <90^\circ$) are less permeable, by a factor of 2 to 6, than those containing oblique or parallel joints (Fig. 3f). The pre-deformation permeability does not vary as a function of joint thickness (Additional file 1).

### Mechanical behaviour

**Fig. 4:**
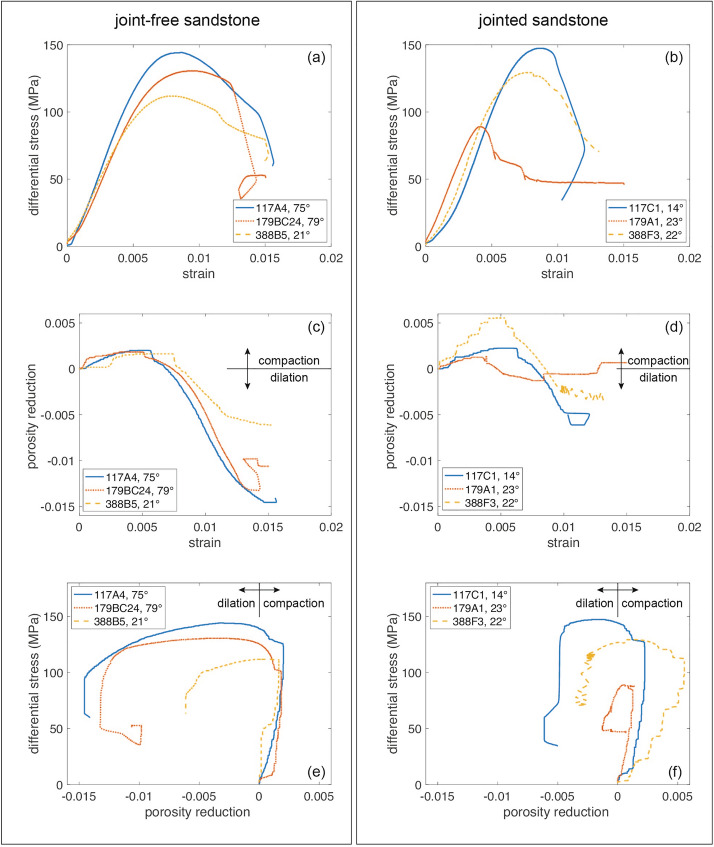
Mechanical data for a selection of joint-free and jointed samples. Left panel: joint-free samples, with oblique bedding. One sample per investigated stratigraphic unit is included. Right panel: jointed samples containing obliquely oriented joints. One sample per investigated stratigraphic unit is included. **a**, **b** Differential stress as a function of strain. **c**, **d** Porosity reduction as a function of strain. **e**, **f** Differential stress as a function of porosity reduction

All samples deformed in this study failed in the brittle regime (Fig. 4a, b; see Additional file 1 for the mechanical data of all samples). In both joint-free and jointed samples, we observe an initial stage of compaction (indicating porosity reduction; Fig. 4), followed by dilatancy (indicating porosity increase; Fig. 4). Peak differential stress decreases as a function of increasing connected porosity and, in jointed samples, does not depend on joint thickness (Additional file 1).

**Fig. 5:**
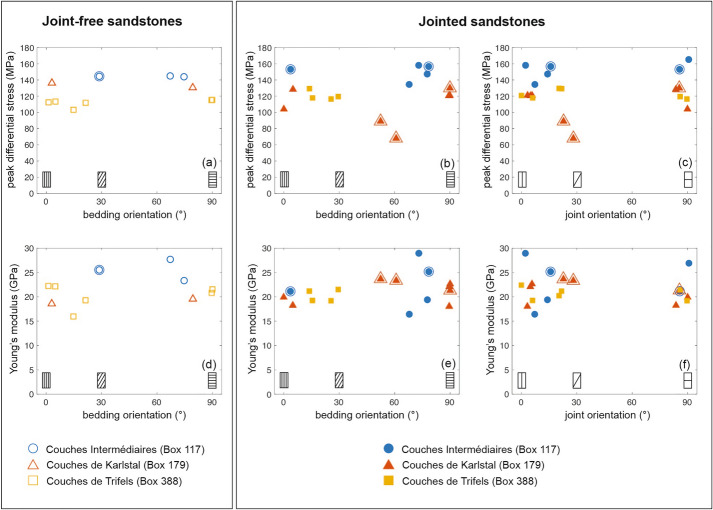
Peak differential stress and Young’s modulus as a function of bedding and joint orientation. Joint-free samples are shown in the left panel (unfilled symbols); jointed samples are shown in the right panel (filled symbols). **a**, **b**, **c** Peak differential stress. **d**, **e**, **f** Young’s modulus. In all panels, circles denote *Couches Intermédiaires* (Box 117) samples; triangles denote *Couches de Karlstal* (Box 179) samples; squares denote *Couches de Trifels* (Box 388) samples. Double-outlined symbols represent samples where the experimentally induced fracture aligns with at least a portion of a pre-existing structural feature. Measurement error is within symbol size

The peak differential stress of joint-free *Couches Intermédiaires* samples is between 144 and 145 MPa; in jointed samples, this value is between 134 and 166 MPa (Table 1). The strength of the joint-free (Fig. 5a, blue circle), and jointed (Fig. 5b, blue circles) samples does not depend on bedding orientation, nor do we observe a relationship between joint orientation and peak differential stress (Fig. 5c, blue circles). Of the nine *Couches Intermédiaires* samples deformed, two jointed samples (117C_2 and 117A_7) and one joint-free sample (117A_8) failed, in part, along their natural structural features (Fig. 2) but none of these samples are weaker than the rest of the sample suite.

Jointed *Couches de Karlstal* samples are consistently weaker than their joint-free counterparts. Joint-free *Couches de Karlstal* samples have a peak differential stress between 130 and 137 MPa; the peak differential stress for jointed samples is between 67 and 129 MPa (Table 1). There is no apparent relationship between bedding orientation and peak differential stress in joint-free (Fig. 5a, red triangles) or jointed samples (Fig. 5b, red triangles). However, jointed samples are weakest when the natural joint is oriented obliquely ($23^\circ <\beta <28^\circ$) to the applied differential stress (Fig. 5c, red triangles) and deformation is localised on the pre-existing joint surface (Fig. 2). These samples broke at small strain, compared to the other samples (Figs. 4b, Additional file 1), and we observe compaction and sliding along the joint surface until the end of the experiment at 0.015 strain (Figs. 4d, Additional file 1).

Joint-free *Couches de Trifels* samples have peak differential stresses between 103 and 116 MPa; peak differential stress of the jointed samples is between 116 and 130 MPa (Table 1). Unlike the *Couches de Karlstal* sandstones, the jointed *Couches de Trifels* samples are consistently stronger than their joint-free counterparts. None of the samples failed on the pre-existing joint surfaces: experimentally induced failure planes either crosscut the pre-existing joints or developed adjacent to them in the rock matrix (Fig. 2). There is no apparent relationship between bedding orientation and peak differential stress in joint-free (Fig. 5a, yellow squares) or jointed (Fig. 5b, yellow squares) samples.

When all samples are taken together, we observe that static Young’s modulus decreases with initial connected porosity (Additional file 1), though no clear trends appear within individual units. Further, there is no discernible relationship between Young’s modulus and feature orientation – be it bedding or joint orientation—in any of the samples (Fig. 5d–f) and Young’s modulus does not depend on joint thickness (Additional file 1).

### Post-deformation permeability

**Fig. 6:**
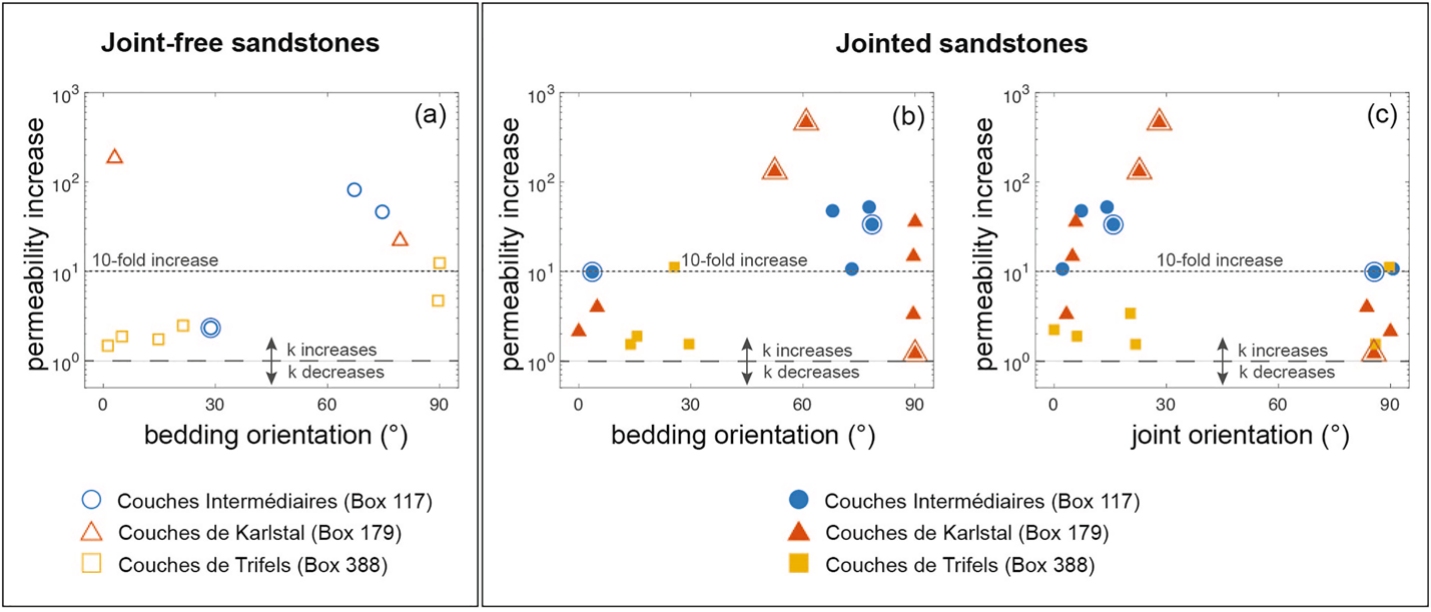
Permeability change as a function of bedding and joint orientation. **a** Permeability change as a function of bedding orientation for joint-free samples. **b**, **c** Permeability change as a function of** b** bedding orientation and** c** joint orientation for jointed samples. Circles denote *Couches Intermédiaires* (Box 117) samples; triangles denote *Couches de Karlstal* (Box 179) samples; squares denote *Couches de Trifels* (Box 388) samples. Unfilled symbols represent joint-free samples and filled symbols represent jointed samples. Double-outlined symbols represent samples where the experimentally induced fracture aligns with at least a portion of a pre-existing structural feature. Measurement error is within symbol size

All samples experienced an increase in permeability after deformation. The post-deformation permeability, *k*_*f*_, of all samples is between 3 × 10^−17^and 1.5 × 10^−13^ m^2^ (Table 1). While *k*_*f*_ does not change systematically as a function of natural feature orientation (Additional file 1), the magnitude of permeability change, ${I}_{k}=\frac{{k}_{f}-{k}_{i}}{{k}_{i}}$, does appear to vary as a function of natural feature orientation (Fig. 6).

Joint-free samples experienced between a 1.2 and 185-fold increase in permeability following deformation (Fig. 6a); jointed samples increased in permeability by a factor of 1.2 to 462 (Fig. 6b, c). For both joint-free and jointed samples, the largest increases in permeability were observed in rocks from the *Couches Intermédiaires* and *Couches de Karlstal* sample suites. There is no systematic relationship between bedding orientation and permeability change in joint-free samples. In jointed samples, permeability change as a function of joint orientation suggests that permeability increased most when joints were oriented between 0 and 30° (Fig. 6c), however, permeability increase diminishes with increasing bedding orientation above $\beta =40^\circ$ (Fig. 6b). *Couches de Trifels* samples experienced the smallest permeability changes and there is no obvious relationship between permeability change and feature orientation in any of these samples.

### Deformation microstructure

**Fig. 7:**
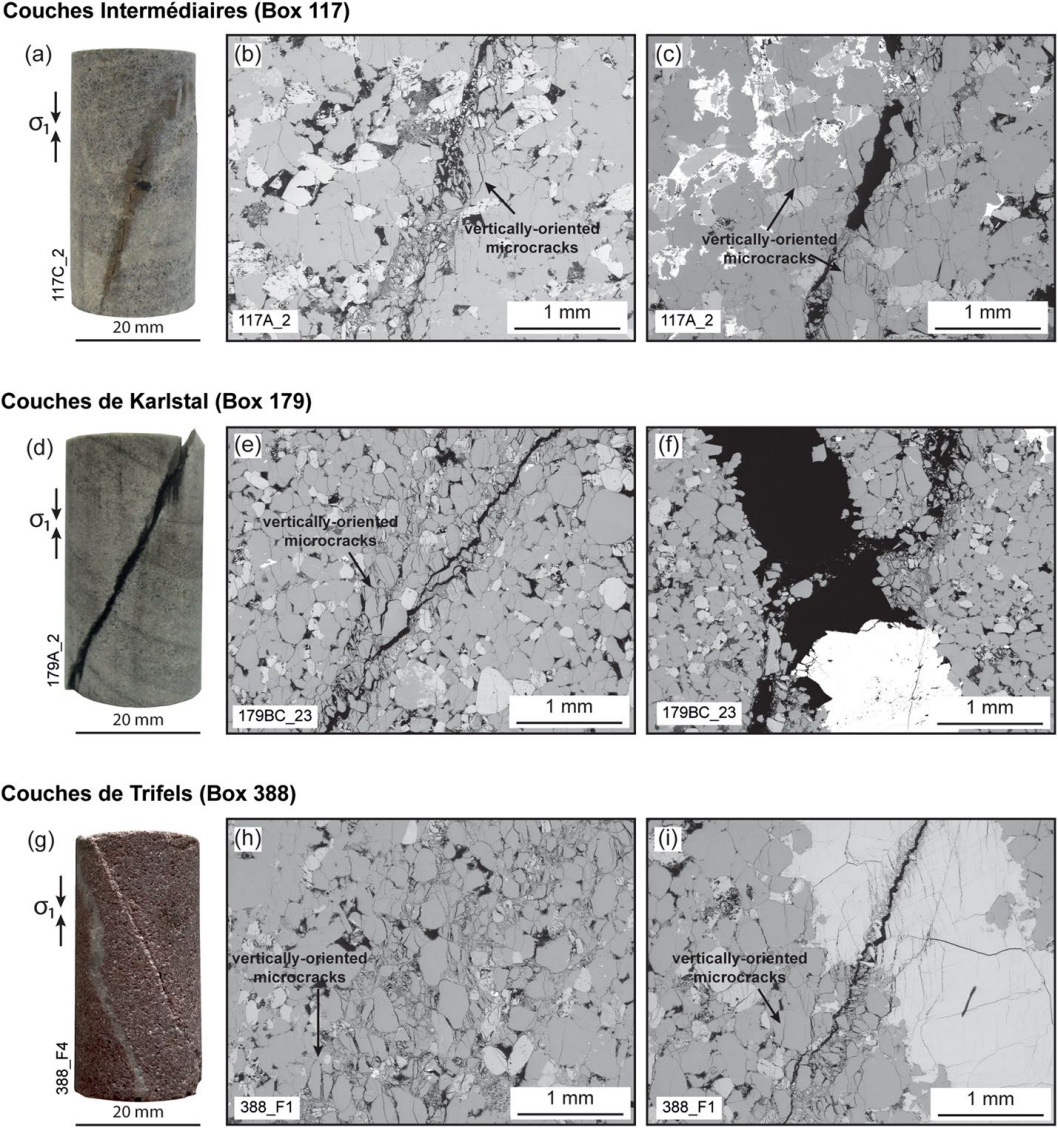
Post-deformation microstructure. Photographs of samples following deformation (**a, d, g**). ${\sigma }_{1}$ denotes the direction of the applied axial load during deformation. Scanning electron microscope (SEM) images showing experimentally induced fractures in the rock matrix (**b**, **e**, **h**) and joints (**c**, **f**, **i**) of the studied sandstones. **a**, **b**, **c** *Couches Intermédiaires* (Box 117); **d**, **e**, **f** *Couches de Karlstal* (Box 179); **g**, **h**, **i** *Couches de Trifels* (Box 388)

The orientations of the experimentally induced failure planes vary from 16 to 39° (Table 1). In most samples, these failure planes manifest as narrow, light-coloured bands (Figs. 2, 7), however, in some samples, failure is clearly visible as a throughgoing fracture on which the sample has broken into two distinct pieces (e.g., 179A_21 and 179A_2, Fig. 2). In thin section, the damage zone within the samples shows shear bands containing crushed grains and microcracks oriented parallel to the loading direction (Fig. 7), which are consistent with the dilation behaviour observed in the pore volume data (Fig. 4).

In the majority of jointed *Couches Intermédiaires* samples, the orientation of the failure plane does not correspond to the orientation of the pre-existing natural feature (Fig. 2). However, strain was partially localized on a pre-existing natural feature in three samples (one joint-free and two jointed samples). In one of these samples (117C_2), the failure plane partially exploits the obliquely oriented joint, but deviates from the joint halfway up the sample. In a second sample (117A_7), the failure plane crosscuts the perpendicularly oriented joint; the failure plane is offset by the joint and the joint in this sample is also broken. The failure plane in a third, joint-free, sample (117A_8) coincides with the obliquely oriented bedding plane.

The experimentally induced failure planes exploit all or a portion of the pre-exiting joints in three *Couches de Karlstal* samples (Fig. 2). Fractures propagated along the entire joint surface in both samples containing obliquely oriented joints (179A_1, 179A_2). In one sample containing a joint oriented perpendicular to the sample length, the failure plane was offset along the joint length and the joint was broken (179BC_25). In a fourth sample containing a joint running parallel to the sample length, the orientation of the fracture was not deviated by the joint, but a second fracture formed in a clay-rich layer (179BC_23); this clay layer was not visible at the sample scale prior to deformation. In all other jointed *Couches de Karlstal* samples, the orientation of the failure planes did not deviate as a result of their interactions with the pre-existing joints and, in SEM, we observe that the fractures cross the joints at locations where there is no joint-filling material (Fig. 7f).

None of the *Couches de Trifels* samples failed along pre-existing natural features (Fig. 2). Indeed, in one sample (388_F4), the fracture formed approximately parallel to the oblique joint surface. We observe no deviation of the failure planes as they cross the joints, but we note that failure plane width decreases where it crosses the joint (Fig. 7i).

## Discussion

### Influence of bedding and variably sealed joints on permeability

The pre-deformation permeability of all joint-free and jointed samples in this study increases with increasing connected porosity (Additional file 1), in agreement with previous observations on sandstones (Bourbié and Zinszner 1985; Nelson 1994; Ehrenberg and Nadeau 2005) and consistent with published data on the Buntsandstein sandstones from the EPS-1 exploratory borehole (Goupil et al. 2022; Griffiths et al. 2016; Heap et al. 2017; Kushnir et al. 2018; Additional file 1). In addition, permeability is anisotropic and largest when bedding is oriented parallel to fluid flow direction (Fig. 3d, e), as previously observed in sandstones (Benson et al. 2003; Louis et al. 2005), including in those from the EPS-1 borehole (Griffiths et al. 2016; Goupil et al. 2022). In the absence of pore shape anisotropy (Louis et al. 2005), permeability anisotropy in the Buntsandstein is the result of bedding-parallel, fine-grained, low-permeability layers (Goupil et al. 2022): when these layers are oriented perpendicular or subperpendicular to fluid flow direction ($\beta >60^\circ$), fluids are obliged to flow through them, and sample permeability approaches that of the low-permeability layers. When these layers are oriented parallel or subparallel to fluid flow direction ($\beta <30^\circ$), fluids can exploit higher-permeability layers.

Although pre-deformation permeability changes as a function of bedding orientation, the influence of joint orientation on pre-deformation permeability is less clear (Fig. 3f). *Couches de Trifels* sandstones are about a factor of two less permeable when joints are oriented perpendicular to the direction of fluid flow: these well-filled joints contain insufficient connected porosity to support fluid flow and act as barriers to flow. However, the permeability of the *Couches Intermédiaires* and the *Couches de Karlstal* rocks is unaffected by joint orientation: the poorly and moderately filled joints in these samples contain sufficient connected porosity to support fluid flow and are neither barriers to nor conduits for fluid flow. The influence, therefore, of a particular joint on permeability depends on the extent of joint sealing (Griffiths et al. 2016), with well-sealed joints acting as low-permeability layers and poorly- to moderately-sealed joints not exerting an important control on permeability at the sample scale.

### Strength, Young’s modulus, and strain localisation

The compressive strength of the rocks studied here decreases as a function of increasing porosity (Additional file 1), consistent with observations on sandstones (e.g., Baud et al. 2014; Chang et al. 2006), including sandstones from the EPS-1 borehole (Heap et al. 2019). With the exception of the *Couches Intermédiaires* sandstones, Young’s modulus also decreases as a function of porosity (Additional file 1), as previously shown for Buntsandstein sandstones (Heap et al. 2019). We do not observe a systematic change in compressive strength nor in Young’s modulus as a function of bedding orientation (Fig. 5), nor do we observe strain localisation on bedding planes (with the exception of sample 117_8), however, bedded sandstones have been previously demonstrated to exhibit mechanical anisotropy. For example, bedded sandstone containing compositionally-defined beds are weaker (Chenevert and Gatlin 1965; Hu et al. 2017) and have lower Young’s modulus (Shi et al. 2020; Yin and Yang 2018) when bedding is oriented 20 − 45° to the applied load. In the absence of obvious compositional variability, grain and/or pore shape anisotropy results in mechanical anisotropy (Louis et al. 2009): sandstones containing non-spherical grains, for example, are weakest when loaded parallel to bedding because the total grain-to-grain contact surface is minimized and the stress at these contacts is, therefore, maximized (Louis et al. 2009). The absence of mechanical anisotropy in our joint-free samples could be because (1) beds are not defined by mechanically weaker minerals, such as clays, and/or (2) grain shape anisotropy within our samples is not sufficiently large enough to exert a discernable control on the mechanical properties of these rocks.

We now assess the role of mineral-filled joints on mechanical behaviour and the propensity for strain localization along these features. Generally, the compressive strength of rocks containing planes of weakness—including compositionally-defined bedding, foliation, and cleavage planes—is lowest when these features are oriented at an oblique angle (~ 20 to 45°) to the applied stress (e.g., Attewell and Sandford 1974; Gottschalk et al. 1990; Nasseri et al. 2003). *Couches de Karlstal* samples containing obliquely oriented joints are notably weaker than the rest of their sample suite (Fig. 5c), consistent with observations of materials containing a single plane of weakness (Jaeger 1960). These samples failed along poorly filled joints whose high porosity makes them locally weaker than the rest of the sample (Fig. 7d). We further note that all jointed *Couches de Karlstal* samples are weaker than their joint-free counterparts regardless of joint orientation, possibly because the presence of localized, high-porosity joints contributes to the overall weakening of the rocks.

By contrast, there is no systematic change in compressive strength as a function of joint orientation in the *Couches Intermédiaires* or the *Couches de Trifels* samples, however, the jointed *Couches de Trifels* samples are stronger than their joint-free counterparts (Table 1). The samples from the *Couches Intermédiaires* and *Couches de Trifels* contain moderately to well-sealed joints, with joint sealing material permeating into the surrounding rock matrix (Fig. 1c, i), reducing porosity and locally increasing rock strength in the vicinity of the joint. Indeed, in one instance (sample 388_F4), the failure plane developed parallel to the joint instead of exploiting it, demonstrating that, despite its optimal orientation, the joint was locally stronger than the surrounding rock matrix. This is consistent with—though opposite to—observations of the strength of stylolite-bearing limestones: porosity increase in proximity to stylolite planes results in the overall reduction in bulk rock strength without any mechanical anisotropy (Baud et al. 2016).

### Shear-enhanced permeability

The permeability of both joint-free and jointed samples in this study increased after deformation (Fig. 6) and our pore volume data (Additional file 1) show dilatancy in all samples during brittle deformation, consistent with previous studies (Read et al. 1995; Wong et al. 1997; Yildirim et al. 2020). Post-deformation microstructure shows shear bands defined by crushed grains and microcracks oriented parallel or subparallel to the loading direction (Horii and Nemat-Nasser 1985; Menéndez et al. 1996) and we conclude that permeability increase following deformation is the result of an interconnected network of dilatant microcracks oriented parallel or subparallel to the direction of fluid flow (Fig. 7). We note that the increase in permeability measured here is less than that observed for experimentally-induced tensile fractures, measured under the same laboratory conditions (Kushnir et al. 2018). One reason for this may be that, unlike the tensile fractures in Kushnir et al. (2018), the fractures in the present study were created in shear and the fracture surfaces do not always connect the top and bottom of the samples (Fig. 2). Further, experimentally induced tensile fractures are open spaces containing little intra-fracture debris, while the permeability of the shear planes produced here is controlled by a more tortuous network of microcracks. Despite these differences in the magnitude of permeability increase, permeability enhancement due to shear fractures is more likely to be sustained under reservoir conditions. Tensile fractures are prone to closing almost entirely at depth, limiting their contribution to permeability enhancement (Yildirim et al. 2020). Indeed, shear displacement along fracture surfaces is key to maintaining enhanced reservoir permeability at depth after the cessation of imposed fluid pressures (Yildirim et al. 2020). Therefore, the permeability changes measured in this study are representative of the potential for permeability enhancement under in situ reservoir conditions.

While experiments have demonstrated that injection-induced shear slip and propagation of open fractures can result in permeability enhancement (Esaki et al. 1999; Park et al. 2013; Ye and Ghassemi 2019), the reactivation of filled fractures is less predictable. In the present study, the greatest increases in permeability are observed in rocks containing poorly to moderately sealed joints that are entirely or partially broken during deformation, thereby reopening these conduits to fluid flow: the largest increases in permeability are observed in *Couches de Karlstal* samples where deformation localised entirely on pre-existing joint surfaces. The smallest permeability increases are observed in *Couches de Trifels* sandstones, where well-sealed joints facilitated neither strain localization nor permeability enhancement. Similarly, fluid pressure injection experiments conducted on a sample of phyllite containing an optimally-oriented calcite vein resulted in neither strain localization nor permeability enhancement on the vein (Ye et al. 2020). While Ye et al. (2020) found that permeability increased by three orders of magnitude, shear did not localize on the vein and, despite the initially higher permeability of the vein compared to the bulk rock, the vein did not contribute to permeability enhancement. We conclude that the propensity for reactivation of sealed joints is strongly dependent not only on the extent to which joints are sealed, but also to the contrast in mechanical properties between the joint-filling material and the host rock.

### Implications for hydraulic stimulation of Enhanced Geothermal Systems

Hydraulic stimulation of Enhanced Geothermal Systems (EGS) can induce shear on pre-existing joints and create new fractures (McClure and Horne 2014), such that the transmissivity of the geothermal reservoir is significantly increased. Hydraulic stimulation is, however, accompanied by an increased risk of anthropogenically induced and/or triggered seismic activity (Deichmann and Giardini 2009; Schmittbuhl et al. 2021; Wassing et al. 2021). Seismicity is an anticipated consequence of movement along planes of weakness in the crust and the creation of new fractures and, therefore, is expected during hydraulic stimulation (Moeck et al. 2009). However, careful design of hydraulic stimulation procedures can reduce the magnitude of induced and/or triggered seismicity. This requires detailed knowledge of the in situ stress state of the system (Cuenot et al. 2006; Cornet et al. 2007), as well as knowledge of the condition of the rock mass being stimulated, including lithology, joint density, joint distribution, joint orientation, and the composition and extent of joint-filling mineralisation (Meller et al. 2012). Slip tendency analysis, for example, uses the geological strength index (GSI) of the reservoir rock mass to account for the weakening presence of joint networks (Moeck et al. 2009).

However, as observed in the present study, the role of joints on the strength and permeability evolution during deformation of Buntsandstein sandstones depends on the degree of fracture filling and the contrast in strength between the joint-filling material and host rock. Poorly sealed joints (e.g., *Couches de Karlstal*) in our study reduce the overall strength of the rock and, when oriented obliquely to the applied stress, act as planes of weakness and reactivated conduits for fluid flow. However, we also find that well sealed joints (e.g., *Couches de Trifels*) locally reinforce and strengthen the sandstone and permeability increase after deformation is modest (up to one order of magnitude). This has profound implications on hydraulic stimulation design. Indeed, hydraulic stimulation of the Poorman Schist formation at the SURF (Stanford Underground Research Facility, USA) testbed found that, on the 10 m scale, natural quartz/calcite-sealed fractures within the phyllite reservoir did not reactivate but instead locally strengthened the rock mass (Fu et al. 2021). Therefore, a rock mass containing a pervasive but well-sealed joint network may require stimulation injection pressures in excess of the minimum local principle stress (McClure and Horne 2014; Li et al. 2022) to create an entirely new network of fractures, while yielding only moderate increases in reservoir permeability. We conclude, therefore, that well sections containing poorly filled joint networks are more favourable targets for hydraulic reservoir stimulation.

## Conclusions

The role of joints on the strength and permeability evolution of Buntsandstein sandstones during deformation depends on the degree of fracture filling. We find that well-sealed joints act as barriers to fluid flow and permeability decreases as joints become oriented perpendicular to fluid flow direction. Well-sealed joints do not reactivate in shear as joint-sealing precipitates reduce porosity not only in the joint, but also in the matrix adjacent to the joint, locally increasing rock strength. While shear failure does increase the bulk permeability of these rocks, permeability enhancement is not facilitated by the joint plane. Poorly sealed joints in this study, on the other hand, act as neither conduits nor barriers to fluid flow prior to deformation, but their increased porosity lends them significant mechanical weakness. These planes of weakness result in lower sample strength with respect to other joint-free samples and, when oriented obliquely to the applied stress, localize strain. The reactivation of these features opens conduits to fluid flow and the post-deformation permeability of these rocks increases significantly, by up to a factor of 500.

Our observations suggest that the degree of joint-filling mineralization and its local mechanical effect are important controls on how easily joints may become reactivated during hydraulic stimulation. Hydraulic stimulation operations need to carefully consider target fracture networks before proceeding: well-sealed rock masses may be stronger than their joint-free counterparts and necessitate injection pressures that exceed local directives and risk producing felt seismic events. In addition to this, once fractured, these rock masses may not yield permeabilities sufficiently high to warrant geothermal exploitation. By targeting poorly sealed fracture networks, hydraulic stimulation may be able to exploit these mechanically weak surfaces using modest injection pressures (i.e., below the minimum principal stress) and significantly increase rock mass permeability, not only increasing the likelihood of developing reservoir permeability to a sufficiently high value, but also decreasing the risk of felt seismic events.

### Supplementary Information

**Additional file 1.** This file contains additional plots referenced to in the main text and complete mechanical and pore volume data.

## Data Availability

All the data collected for this study can be found in Table 1 and in the Additional file 1.
