# Supplementary material for: Reactivation of variably sealed joints and permeability enhancement in geothermal reservoir rocks
Source: Geotherm Energy (Heidelb). 2023 Nov 13;11(1):32. doi: 10.1186/s40517-023-00271-5 (PMC10658799; doi:10.1186/s40517-023-00271-5)
Supplement: Supplementary file 1 — Additional file 1. This file contains additional plots referenced to in the main text and complete mechanical and pore volume data. [file 40517_2023_271_MOESM1_ESM.docx]

**Additional files to:**

**Reactivation of variably sealed joints and permeability enhancement in geothermal reservoir rocks**

**Authors:**

Kushnir, Alexandra R. L. ^a, b*^

Heap, Michael J. ^a, c^

Baud, Patrick ^a^

Thierry Reuschlé ^a^

Jean Schmittbuhl ^a^

^a^ *Université de Strasbourg, CNRS, Institut Terre et Environnement de Strasbourg, UMR 7063, 5 rue René Descartes, Strasbourg F-67084, France*

^b^ now at : *Rock Physics and Geofluids Laboratory (RPGL), École Polytechnique Fédérale de Lausanne, Lausanne, Switzerland*

^c^ *Institut Universitaire de France (IUF), Paris, France*

**Contents :**

**S1. Pre-deformation permeability as a function of connected porosity**

**S2. Mechanical and porosity reduction data for all samples**

**S3. Peak differential stress and Young’s modulus as a function of connected porosity**

**S4. Connected porosity, permeability, peak differential stress, and Young’s modulus as a function of joint thickness**

**S5. Post-deformation permeability as a function of bedding and joint orientation**

**S6. Data compilation of pre-deformation permeability as a function of connected porosity for Buntsandstein from the EPS-1 borehole**

**S1. Pre-deformation permeability as a function of connected porosity**


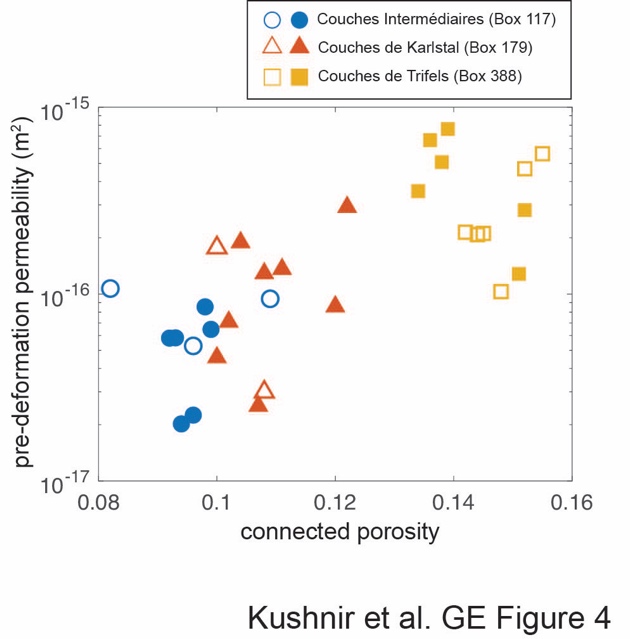


**Figure S1. Pre-deformation permeability of the Buntsandstein samples as a function of connected porosity.** Unfilled symbols are joint-free samples; filled symbols are jointed samples. Circles denote *Couches Intermédiaires* (Box 117) samples; triangles denote *Couches de Karlstal* (Box 179) samples ; squares denote *Couches de Trifels* (Box 388) samples. Measurement error is within symbol size.

**S2. Mechanical and porosity reduction data for all samples**


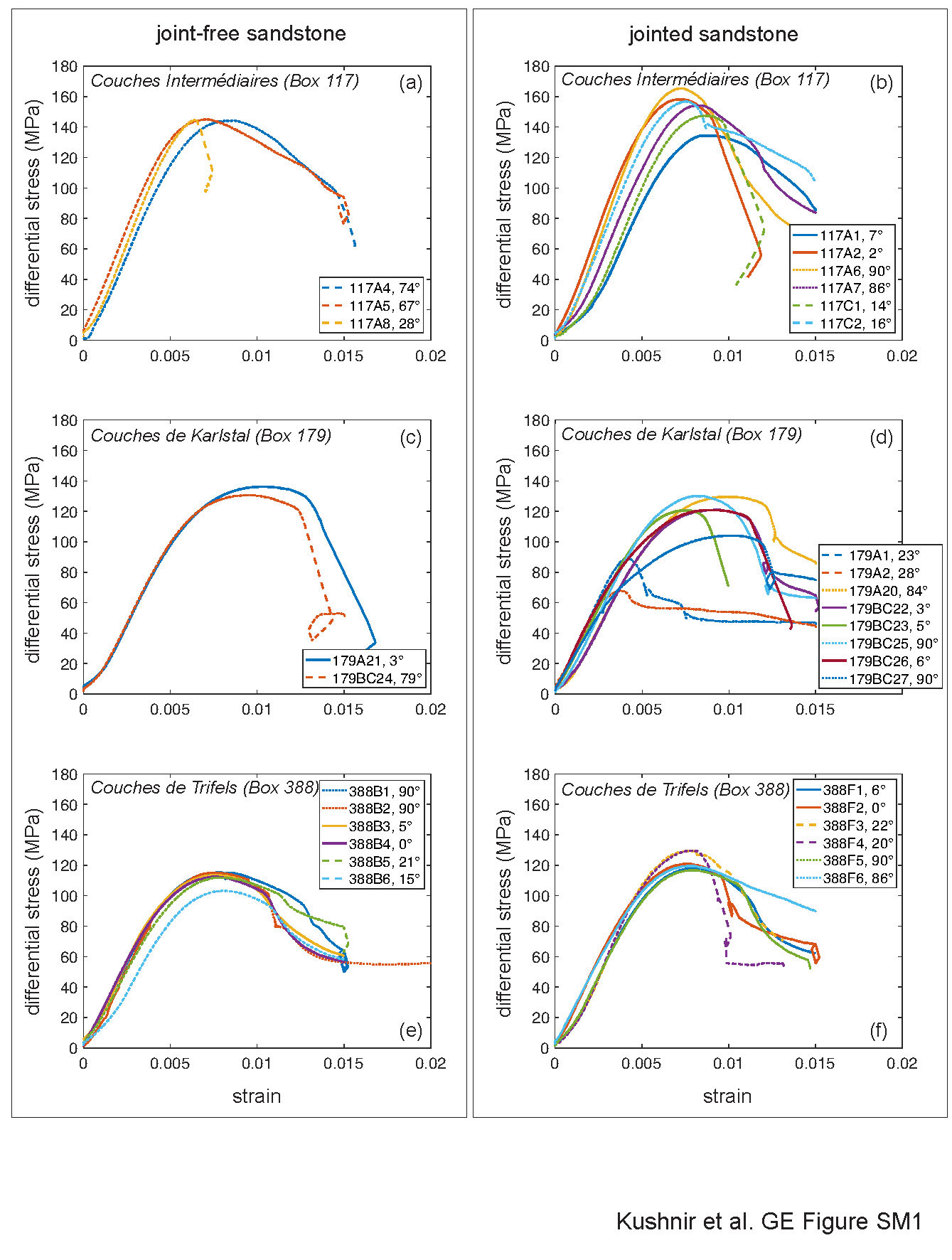


**Figure S2. Differential stress as a function of strain. (a, b)** *Couches Intermédiaires.* **(c, d)** *Couches de Karlstal*. **(e, f)** *Couches de Trifels.* Left panel shows data for joint-free samples; right panel shows data for jointed samples. For all figures: solid lines denote samples with bedding/joints parallel to the applied stress; dashed lines denote samples with bedding/joints oriented obliquely to the applied stress; and dotted lines denote samples with bedding/joints oriented perpendicular to the applied stress.


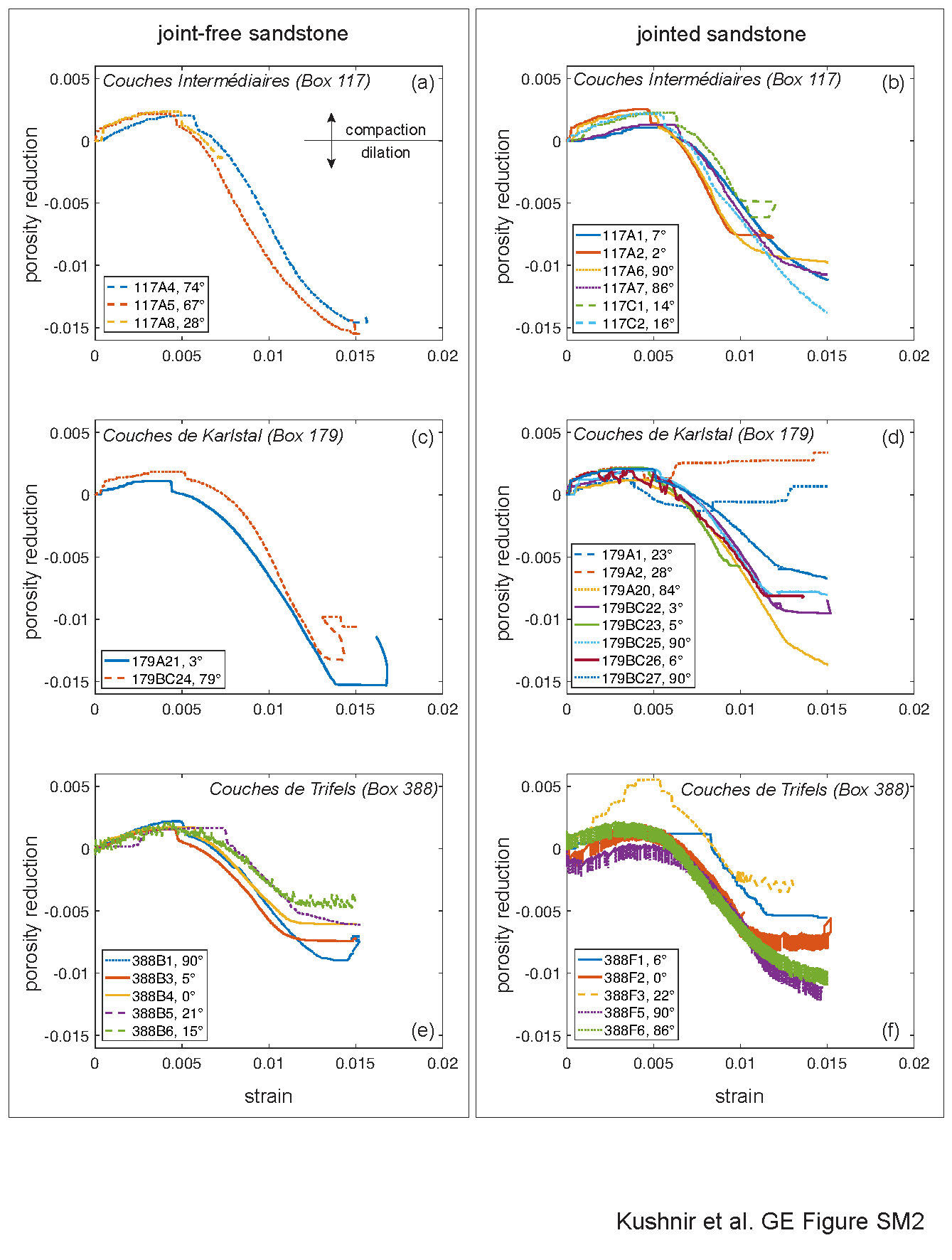


**Figure S3. Porosity reduction as a function of strain. (a, b)** *Couches Intermédiaires.* **(c, d)** *Couches de Karlstal*. **(e, f)** *Couches de Trifels*; data for samples 388_B2 and 388_F4 have been excluded due to problems encountered by the pore fluid pressure encoder during deformation*.* Left panel shows data for joint-free samples; right panel shows data for jointed samples. For all figures: solid lines denote samples with bedding/joints parallel to the applied stress; dashed lines denote samples with bedding/joints oriented obliquely to the applied stress; and dotted lines denote samples with bedding/joints oriented perpendicular to the applied stress.


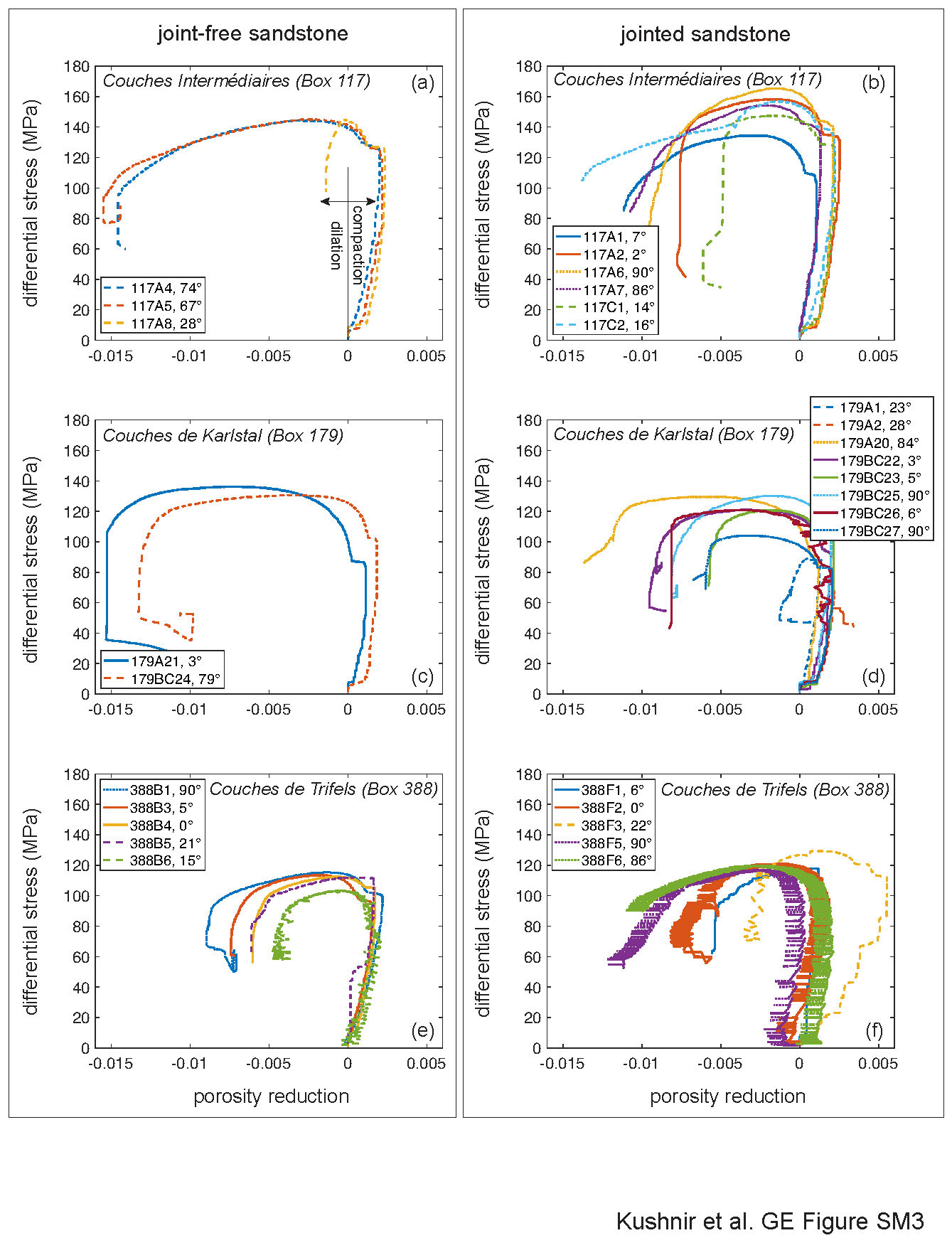


**Figure S4. Differential stress as a function of porosity reduction. (a, b)** *Couches Intermédiaires.* **(c, d)** *Couches de Karlstal*. **(e, f)** *Couches de Trifels*; data for samples 388_B2 and 388_F4 have been excluded due to problems encountered by the pore fluid pressure encoder during deformation*.* Left panel shows data for joint-free samples; right panel shows data for jointed samples. For all figures: solid lines denote samples with bedding/joints parallel to the applied stress; dashed lines denote samples with bedding/joints oriented obliquely to the applied stress; and dotted lines denote samples with bedding/joints oriented perpendicular to the applied stress.

**S3. Peak differential stress and Young’s modulus as a function of connected porosity**


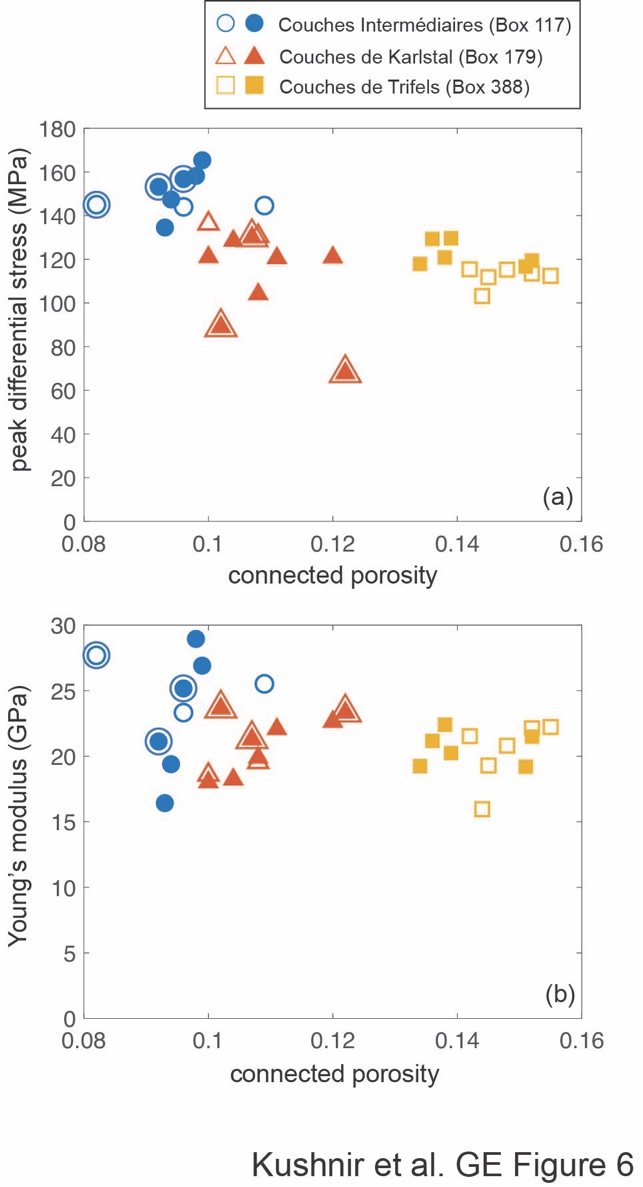


**Figure S5. Peak differential stress (a) and Young’s modulus (b) as a function of connected porosity.** Unfilled symbols represent joint-free samples; filled symbols represent jointed samples. Circles denote *Couches Intermédiaires* (Box 117) samples ; triangles denote *Couches de Karlstal* (Box 179) samples ; squares denote *Couches de Trifels* (Box 388) samples. Double-outlined symbols represent samples where the experimentally induced fracture aligns with at least a portion of a pre-existing structural feature. Measurement error is within symbol size.

**S4. Connected porosity, permeability, peak differential stress, and Young’s modulus as a function of joint thickness**


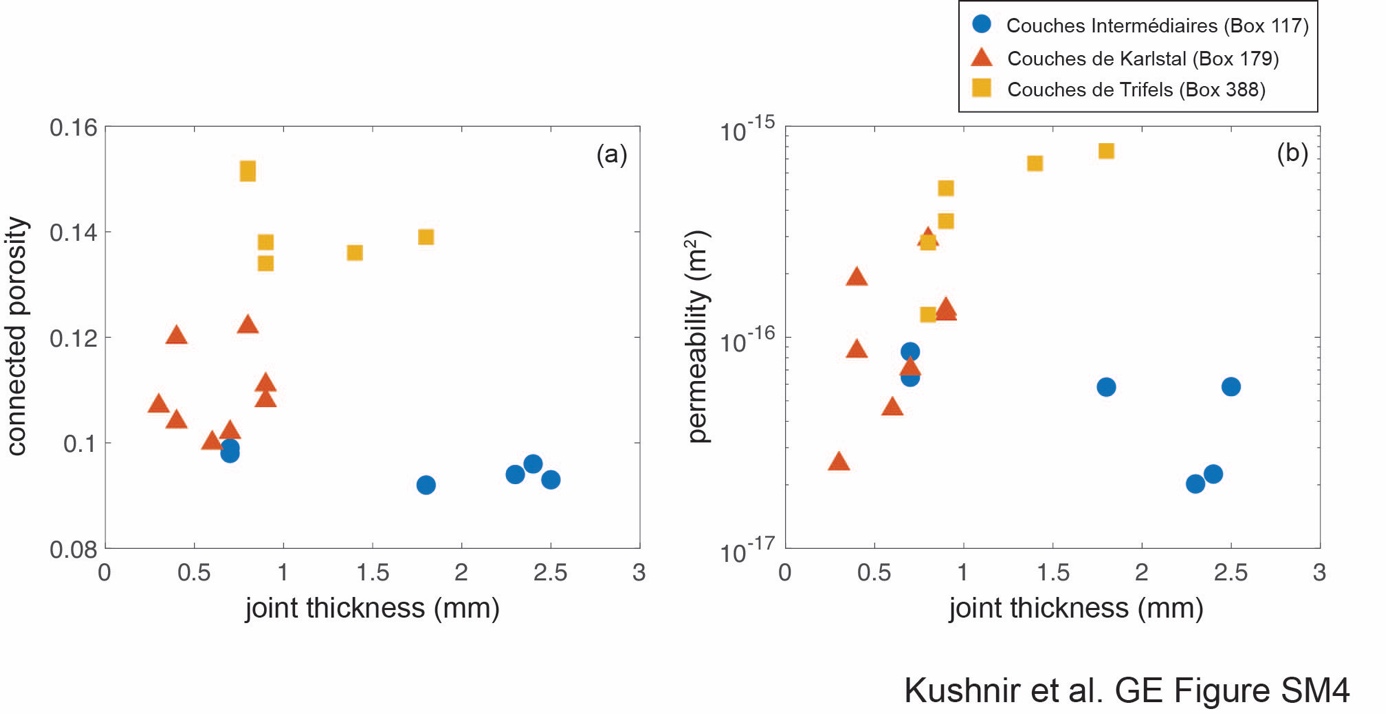


**Figure S6. Connected porosity and permeability as a function of average joint thickness, before deformation.** **(a)** Connected porosity. **(b)** Permeability. In all figures, circles denote *Couches Intermédiaires* (Box 117) samples; triangles denote *Couches de Karlstal* (Box 179) samples; squares denote *Couches de Trifels* (Box 388) samples. Measurement error is within symbol size.


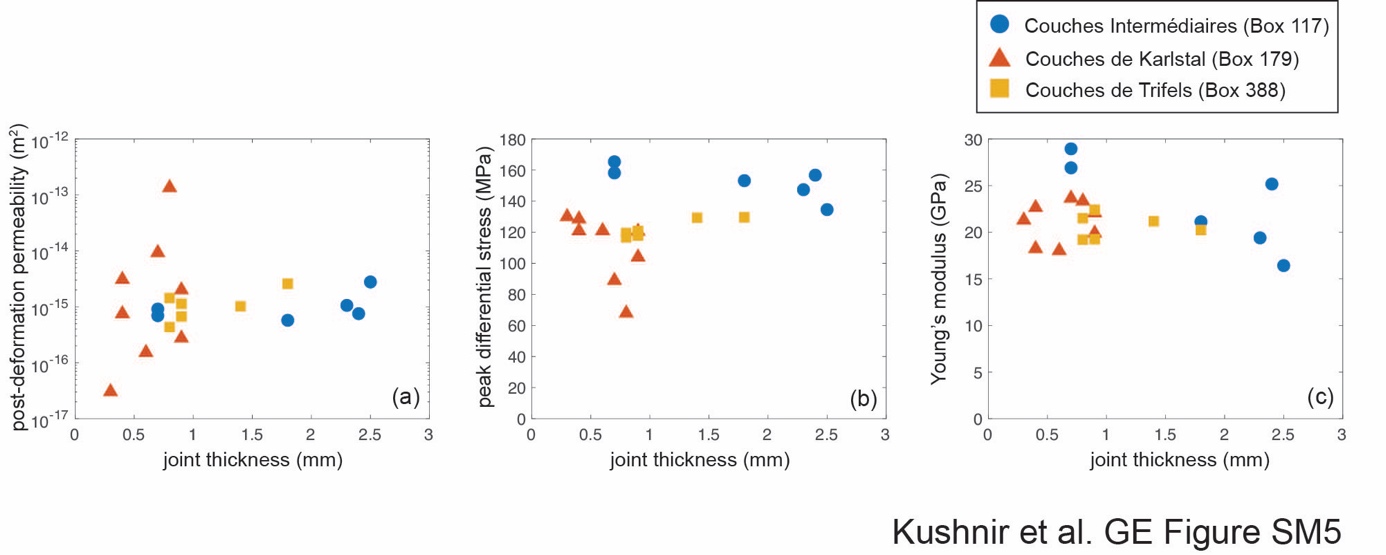


**Figure S7. Post-deformation permeability, peak differential stress, and Young’s modulus as a function of average joint thickness, before deformation.** **(a)** Post-deformation permeability. **(b)** Peak differential stress. **(c)** Young’s modulus. In all figures, circles denote *Couches Intermédiaires* (Box 117) samples; triangles denote *Couches de Karlstal* (Box 179) samples; squares denote *Couches de Trifels* (Box 388) samples. Measurement error is within symbol size.

**S5. Post-deformation permeability as a function of bedding and joint orientation**


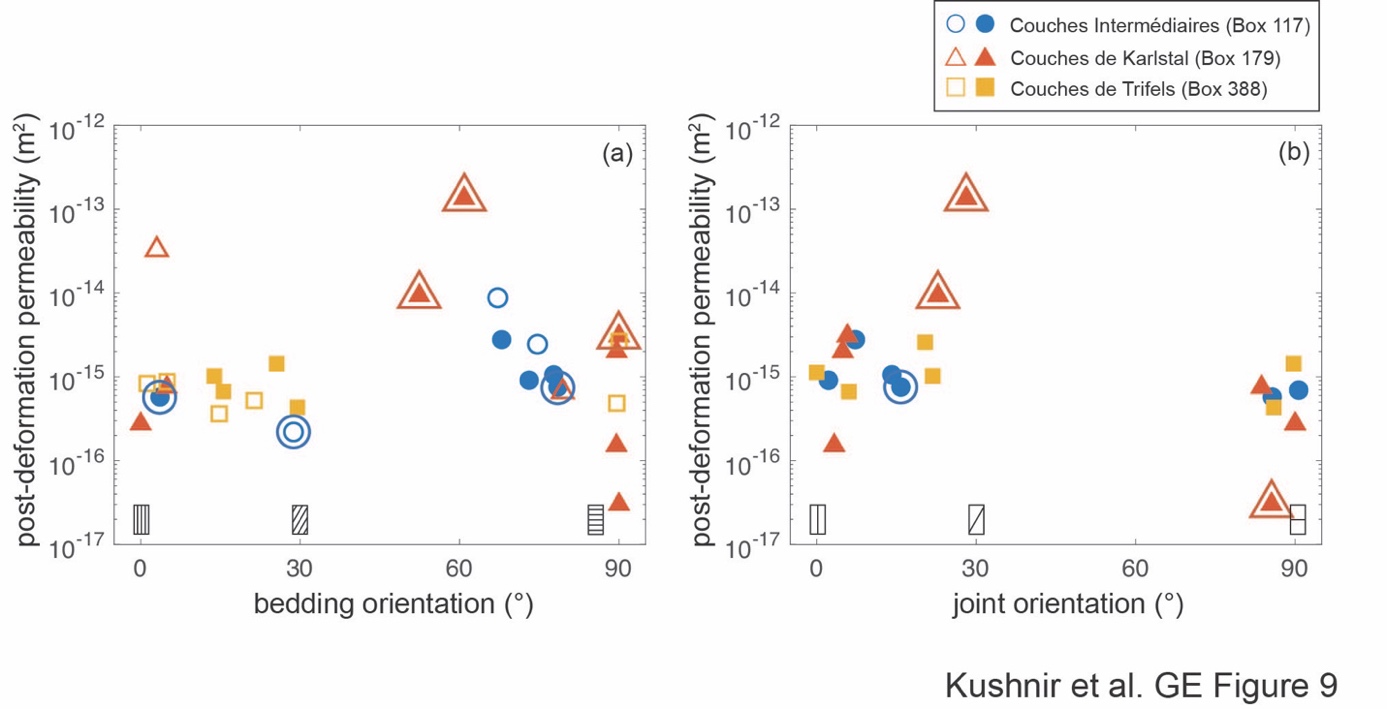


**Figure S7. Post-deformation permeability of joint-free and jointed samples as a function of (a) bedding orientation and (b) joint orientation.** Circles denote *Couches Intermédiaires* (Box 117) samples; triangles denote *Couches de Karlstal* (Box 179) samples; squares denote *Couches de Trifels* (Box 388) samples. Unfilled symbols represent joint-free samples; filled symbols represent jointed samples. Double-outlined symbols represent samples where the experimentally induced fracture aligns with at least a portion of a pre-existing structural feature. Measurement error is within symbol size.

**S6. Data compilation of pre-deformation permeability as a function of connected porosity for Buntsandstein from the EPS-1 borehole**


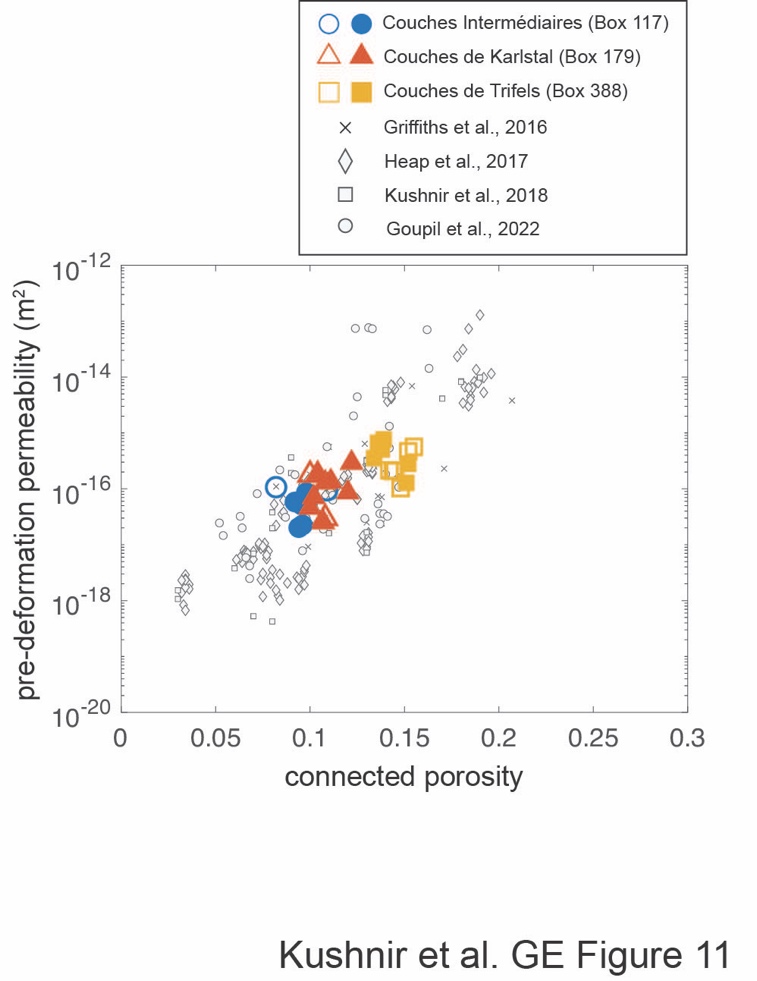


**Figure S9. Data compilation of pre-deformation permeability as a function of connected porosity for Buntsandstein from the EPS-1 borehole.** Data include the samples in the present study as well as data gathered by (Griffiths et al. 2016; Heap et al. 2017; Kushnir et al. 2018; Goupil et al. 2022).
